# Bio-Inspired Microwave Modulator for High-Temperature Electromagnetic Protection, Infrared Stealth and Operating Temperature Monitoring

**DOI:** 10.1007/s40820-021-00776-3

**Published:** 2021-12-13

**Authors:** Xuan Yang, Yuping Duan, Shuqing Li, Huifang Pang, Lingxi Huang, Yuanyuan Fu, Tongmin Wang

**Affiliations:** 1 Key Laboratory of Solidification Control and Digital Preparation Technology (Liaoning Province), School of Materials Science and Engineering, Dalian University of Technology, Dalian, 116085 People’s Republic of China; 2 Science and Technology On Power Beam Processes Laboratory, AVIC Manufacturing Technology Institute, Beijing, 100024 People’s Republic of China

**Keywords:** Microwave modulator, Electromagnetic protection, High temperatures, Temperature monitoring, Carbonized wood

## Abstract

High-temperature electromagnetic (EM) protection materials integrated of multiple EM protection mechanisms and functions are regarded as desirable candidates for solving EM interference over a wide temperature range. In this work, a novel microwave modulator is fabricated by introducing carbonyl iron particles (CIP)/resin into channels of carbonized wood (C-wood). Innovatively, the spaced arrangement of two microwave absorbents not only achieves a synergistic enhancement of magnetic and dielectric losses, but also breaks the translational invariance of EM characteristics in the horizontal direction to obtain multiple phase discontinuities in the frequency range of 8.2–18.0 GHz achieving modulation of reflected wave radiation direction. Accordingly, CIP/C-wood microwave modulator demonstrates the maximum effective bandwidth of 5.2 GHz and the maximum EM protection efficiency over 97% with a thickness of only 1.5 mm in the temperature range 298–673 K. Besides, CIP/C-wood microwave modulator shows stable and low thermal conductivities, as well as monotonic electrical conductivity-temperature characteristics, therefore it can also achieve thermal infrared stealth and working temperature monitoring in wide temperature ranges. This work provides an inspiration for the design of high-temperature EM protection materials with multiple EM protection mechanisms and functions.

**Supplementary Information:**

The online version contains supplementary material available at 10.1007/s40820-021-00776-3.

## Highlights

- A multifunctional microwave modulator is developed for electromagnetic protection, infrared stealth and operating temperature monitoring over wide temperature ranges for the first time.
- Microwave modulator achieves the integration of two electromagnetic protection mechanisms, microwave absorption and radiation deflection.
- Microwave modulator demonstrates the maximum effective bandwidth of 5.2 GHz with a thickness of only 1.5 mm in the temperature range 298–673 K.

**Supplementary Information:**

The online version contains supplementary material available at 10.1007/s40820-021-00776-3.

## Graphical abstract

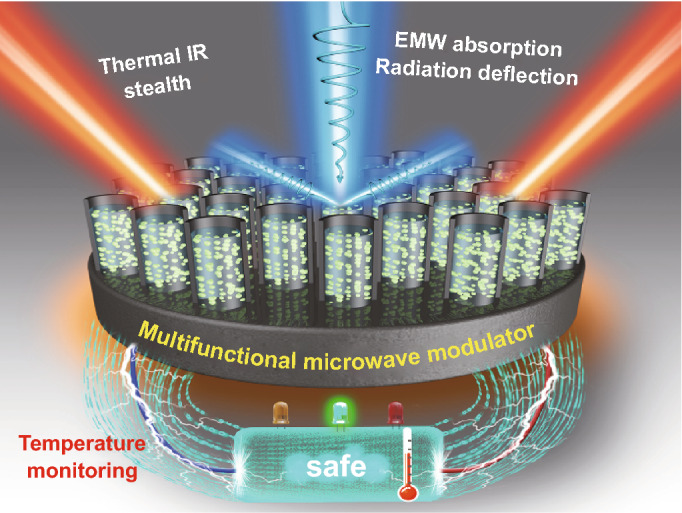

## Introduction

With the development of various high-powered electronic devices and pulse weapons, electromagnetic (EM) radiation and thermal impact often present simultaneously, and thus EM interference in a wide temperature range has become new challenge for EM protection [1, 2]. In addition, temperature differences between devices and background caused by the dynamic working conditions are more likely to render the protected target visible to infrared detector, and wide operating temperature ranges also pose higher requirements for the reliability of EM protection materials [3, 4]. Therefore, faced with a complex and dynamic application environments, it is necessary for the next-generation of EM protection materials to further improve EM protection capacity and to be able to cope with some temperature-induced derivative problems.

High-temperature microwave absorbing materials, as an effective means of providing EM protection in a wide temperature range, have been receiving great attention [5]. Many researchers have also enhanced the performance of various high-temperature microwave absorbents by the ingredient-morphology synergy. Cao et al. demonstrated a three-dimensional (3D) nets constructed by nickel chains. Due to construction with well-defined conductive paths, temperature-driven conductance loss increases markedly, and 3D nets show an effective bandwidth over 3 GHz at 573 K with a thickness of 1.8 mm [6]. The grape-like Fe_3_O_4_-multiwalled carbon nanotubes composites are synthesized by Yuan et al. Unique multiscale-assembled morphology builds a large number of interfaces for polarization loss, achieving an effective bandwidth in X-band in the temperature range of 323–473 K with a thickness of 3.2 mm [7]. Besides, many hybrid absorbents with fibrous-like or core–shell structures also exhibit enhanced microwave absorption properties at elevated temperatures [8, 9]. However, the morphological designs for absorbents are essentially an enhancement of the intrinsic EM loss mechanisms including magnetic and dielectric losses. In other words, the vast majority of current high-temperature microwave absorbing materials still rely solely on the intrinsic EM loss mechanisms of absorbents to achieve EM protection, which also makes it difficult for high-temperature microwave absorbing materials to achieve further breakthroughs in effective bandwidth and thickness.

According to the EM protection principles, in addition to EM loss mechanisms, regulation of radiation direction for reflected wave is also an effective means to solve problems of EM interference and EM stealth failure in high-temperature environments [10, 11]. Thus, based on current high-temperature microwave absorbing materials, the development of a novel high-temperature EM protection material integrating above two EM protection mechanisms facilitates the simultaneous optimization of effective bandwidth and thickness. In accordance with electromagnetic transmission theory [10, 12], deflection of reflected wave is a result of superposition for multiple electromagnetic wave (EMW) with different phases. Obviously, general microwave absorbing materials obtained by uniformly dispersing absorbents in matrices have translational invariance of EM properties in the horizontal direction, failing to obtain EMW with different phases. Therefore, it is necessary to design a reasonable spatial configuration for absorbents to break translational invariance of EM properties. Many creatures in nature have given us important inspiration in spatial configuration design, such as *Diphylleia grayi*, *Charidotella egregia* and *Dynastes hercules* having evolved nano/micropore arrays which can be filled with different media (water or air) depending on the external humidity [13–15]. Nano-/micropores filled with different media have different refractive index differences with neighboring matrices, which achieves the switch between light refraction and backward scattering. This interesting example inspires us that in the microwave band, spaced arrangement of absorbents with different EM characteristics may break the translational invariance, achieving modulation of the transmission properties of EMW.

Herein, we develop a novel microwave modulator for wide-temperature EM protection by vacuum impregnation of carbonyl iron particles (CIP)/resin into channels of carbonized wood (C-wood). Compared to conventional high-temperature microwave absorbing materials, the spaced arrangement of two absorbents with significant differences in EM parameters breaks the translational invariance of EM characteristics, achieving the modulation of radiation direction of EMW. Due to the integration of both microwave absorption and radiation deflection electromagnetic protection mechanisms, CIP/C-wood microwave modulator with a thickness only 1.5 mm shows a superior EM protection capacity in the temperature range of 298–673 K, the maximum effective bandwidth of 5.2 GHz and the maximum EM protection efficiency over 97%. Moreover, CIP/C-wood microwave modulator exhibits monotonic electrical conductivity-temperature property and low thermal conductivity, and it therefore can be used not only as a temperature sensor to monitor the operating temperature but also as an infrared stealth sheet to achieve thermal camouflage. This multifunctional microwave modulator provides an effective solution to the issues of EM interference in complex environments.

## Experimental Sections

### Materials

*Dynastes Hercules* specimen was provided by Liaoning Entomological Museum, China. Basswood blocks were provided by Henan Wood Company. CIP were purchased from Jiangsu Tianyi Company. Epoxy resin was fabricated by Tetrachem Company. Curing agent, methylhexahydrophthalic anhydride (MHHPA), was provided by Shenchuang Chemical Company. The γ-Aminopropyl triethoxysilane (KH550) was from Jiangsu Chenguang Company.

### Preparation of C-Wood and CIP/C-Wood

The basswood blocks were cut into slices with a thickness of 3.5 mm along the radial direction, and obtained wood slices were pre-oxidized at 533 K for 6 h. After pre-oxidation, the wood slices were carbonized in Ar at 948 K for 2 h with a heating rate of 5 K/min. The C-wood slices were carefully polished with 2000 grit sandpaper to obtain a thickness of 1.5 mm. Homemade molds were used to control the length and width of C-wood to 15 mm. Residual carbon was removed by ultrasonic washing. Then, surface treatment of C-wood was conducted by KH550 (15 wt%). Finally, dry the C-wood at 383 K for 6 h.

CIP/resin composite was obtained by mixing CIP with epoxy resin, as well as MHHPA in proportion (CIP/epoxy resin/MHHPA = 2.3:0.4:0.6, mass ratio). The C-wood was soaked in CIP/resin composite under vacuum for 8 h. Remove excess CIP/resin from the surface of C-wood and then the CIP/C-wood composites were cured at 393 K in vacuum for 2 h. Finally, CIP/C-wood composites were stabilized in Ar at 773 K for 2 h.

### Characterization

Scanning electron microscopy (SEM) images were recorded by SUPRA55 SEM equipped with energy-dispersive X-ray spectrum. Thermo-oxidative stability of specimens was characterized by thermogravimetric analysis (TGA851e). Phase analysis of samples was performed by X-ray diffraction spectrum obtained from X-ray diffractometer (XRD, Empyrean, Co Kα) scanning in 2*θ* range from 10° to 90°. The electrical conductivity of CIP/C-Wood was obtained from an in situ variable temperature electrical conductivity test system in the temperature range of 298–673 K. Fourier Transform Infrared (FTIR) spectra were recorded on a TSS-5X spectrometer (Japan) in the range of 400 to 4000 cm^−1^ to determine the functional groups of C-wood, C-wood-KH550, and epoxy resin. Thermal conductivity of specimens at different temperatures was characterized by Laser Thermal Conductivity Analyzer (NETZSCH-LFA). A high-temperature wave guide test system was used to record the electromagnetic parameters of specimens. The electromagnetic protection properties of C-wood and CIP/C-wood at different temperatures were characterized by arch method in a microwave anechoic chamber. The corresponding electromagnetic protection efficiency of samples can be calculated by Eq. (1):

1 $${\text{RL}} = 10\log \frac{{P_{{\text{R}}} }}{{P_{{\text{I}}} }}$$

where RL, *P*_R_ and *P*_I_ represent reflection loss, reflected powder and incident power of EM wave, respectively.

### Simulation

A software, ANSYS high frequency structure simulator (HFSS), was used to build models and analyze electromagnetic field related issues. According to the SEM images of C-wood and CIP/C-wood, we abstracted a square-ring array as the base structural unit. The inner edge length and outer edge length of square ring are 30 and 40 μm. The excitations and boundaries of model are the Floquet port excitation, as well as Master and Slave boundaries, respectively. Electromagnetic parameters for simulation have been measured by a high-temperature wave guide test system. Then, the far-field radiation maps, phase diagrams and electromagnetic microwave volume loss density can be obtained by carrying out the calculations.

## Results and Discussion

### Inspiration for Spatial Configuration Design

**Fig. 1:**
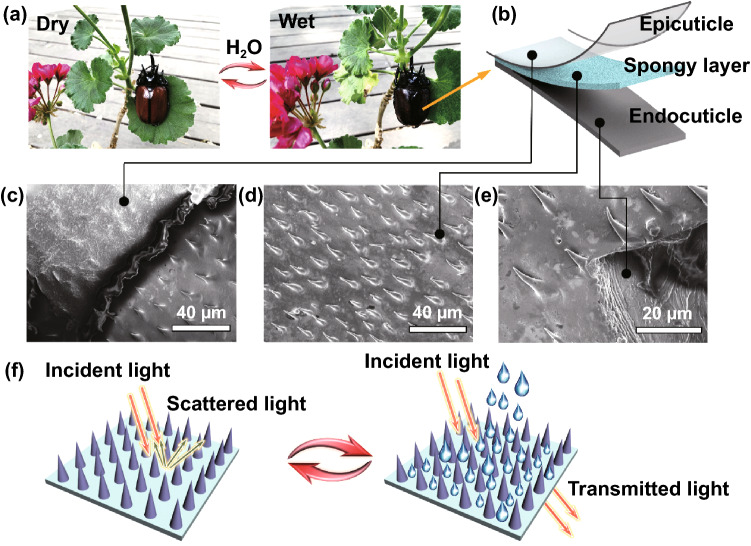
**a** Digital images for elytra color varying with environmental humidity. **b** A schematic of elytra structure. **c** SEM image of epicuticle. **d** SEM image of spongy layer. **e** SEM image of endocuticle. **f** Schematic diagram for humidity changing the path of light transmission in elytra

In this part, inspiration for spatial configuration design is elucidated by an interesting example of dynamic structural color variation in beetle elytra. After a long period of natural selection, Hercules beetles, *Dynastes hercules*, have evolved delicate structure that can modulate their elytra color in response to changes in the external environment [16]. As ambient humidity increases, the color of elytra changes from light brown to dark brown, and elytra would change back to light brown when the environment becomes dry (Fig. 1a). In fact, elytra of beetle is a three-layer structure, including epicuticle, spongy layer and endocuticle (Fig. 1b). As shown in Fig. 1c, epicuticle of elytra is flat without special structure, and there are studies demonstrated that epicuticle has good light permeability [14]. Under the epicuticle, there are a large number of spike-like projections (10–20 μm) on the surface of spongy layer, which leaves a gap of several tens of microns between spongy layer and epicuticle (Fig. 1d). As shown in Fig. 1e, the bottom of spongy layer is closely connected to the opaque endocuticle. In a dry environment, air fills the gap between epicuticle and spongy layer. According to the light transmission theory [17], due to a large difference in refractive index between air (refractive index, *n* = 1) and surrounding spike-like projections, incident light would be backscattered on the surface of sponge layer, thus elytra appearing light brown [18, 19]. On rainy days, water (refractive index, *n* = 1.333) gradually fills the gap, which reduces the refractive index difference with surrounding spike-like projections. Hence, backward scattered light is converted to transmitted light and the dark brown endocuticle of elytra is seen (Fig. 1f). By changing the difference in refractive index between two adjacent materials, beetles can effectively modify the propagation direction of light in elytra, achieving dynamic structural color variation. Similarly, in the microwave band, if we arrange two materials with different EM parameters at intervals, we may be able to optimize the radiation direction of EMW, thus obtaining a novel structural EM protection mechanism.

### Preparation of CIP/C-Wood Microwave Modulator

**Fig. 2:**
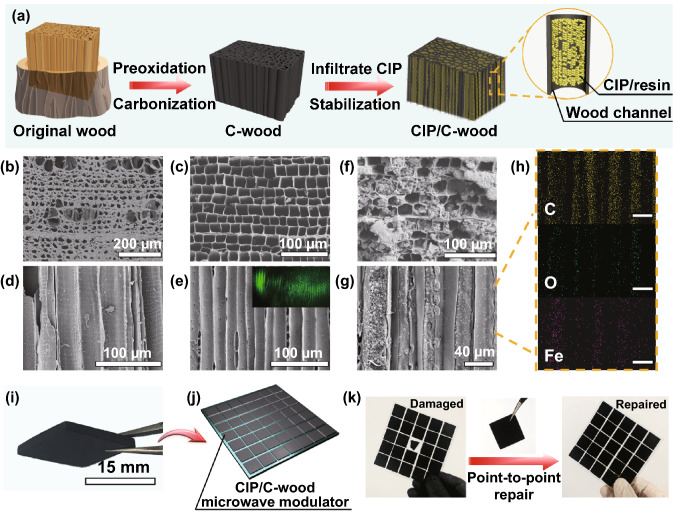
**a** Schematic of the preparation of CIP/C-wood. **b** Cross-sectional SEM image of original wood (basswood). **c** Cross-sectional SEM image of C-wood. **d** SEM image of original wood in the longitudinal direction. **e** SEM image of C-wood in the longitudinal direction. The inset is a scattering pattern of transmitted laser through C-wood. **f** Cross-sectional SEM image of CIP/C-wood. **g** SEM image of CIP/C-wood in the longitudinal direction. **h** Elemental mappings of CIP/C-wood. **i** A digital image of CIP/C-wood. **j** Schematic of CIP/C-wood microwave modulator. **k** Schematic of point-to-point repair

Wood is a ubiquitous structural material with naturally abundant, aligned longitudinal cells and is widely used in energy storage, thermal insulation, EM protection and optical devices, etc. [20–22]. Herein, the basswood with a large number of well-aligned channels is selected as template for the construction of spaced arrangement configuration. As illustrated in Fig. 2a, natural basswood is cut into slices with a thickness of 3.5 mm along the radial direction, and obtained wood slices are pre-oxidized at 533 K for 6 h, followed by carbonization in Ar at 948 K for 2 h. As shown in the top-view SEM images of original wood and C-wood, despite shrinkage of basswood in the radial direction after carbonization, the multi-channel structure of basswood is completely retained with diameters about 20–40 µm, and these channels become more organized (Fig. 2b, c). Longitudinal section views show that wood channels are highly aligned straight before and after carbonization (Fig. 2d, e). When C-wood is vertically irradiated by a laser with wavelength of 532 nm, the grid-like scattering patterns further illustrate the desirable collimation and penetration of C-wood channels (inset of Fig. 2e). In the Raman spectrum of C-wood (Fig. S1a), peak intensity ratio of D band to G band is 1.02, which indicates that a certain amount of more stable graphitization areas appear in the C-wood. Therefore, C-wood exhibits an excellent thermal stability, oxidation temperature over 673 K (Fig. S1b). In order to get better infiltration effect, surface treatment of C-wood is conducted using silane coupling agent (KH550). As shown in Fourier transform infrared (FTIR) spectra (Fig. S2), Si–O–C vibrated at wavelength of 1120 cm^−1^ is the reaction product between the –Si–OH from KH550 and the –OH from C-wood [23]. Absorption peaks corresponding to –CH_3_ and –CH_2_– stretching vibration located at 2926 and 2860 cm^−1^ are observed in FTIR spectrum of C-wood-KH550, demonstrating a successful grafting of coupling agent to C-wood [24, 25]. C-wood-KH550 and epoxy resin have the same chemical groups, which facilitates full impregnation of CIP/resin in C-wood channels. After vacuum impregnation and stabilization, C-wood channels are filled with CIP/resin (Fig. 2f, g), and the uniform distribution of Fe, O, and C elements indicates that there is no significant concentration gradient of CIP/resin in C-wood channels (Fig. 2h). According to results of thermogravimetric analysis, as well as X-ray diffraction, CIP/C-wood shows an excellent thermo-oxidative stability (Fig. S3). On the one hand, a large number of well-aligned channels provide protective barriers for CIP, effectively reducing the area exposed to air. On the other hand, epoxy resin shrinks centripetally with CIP as the core to form a tight cladding layer during the stabilization process (Fig. S4 and S5). Besides, as depicted in Figs. 2i, j and S6a, CIP/C-wood can be cut into various desirable size, as well as shapes, and then arranged on substrates as microwave modulator to provide EM protection. The flexible and versatile shapes of CIP/C-wood microwave modulator also facilitate point-to-point precision repair, reducing maintenance costs and time (Fig. 2k).

### Integration of EM Protection Mechanisms

**Fig. 3:**
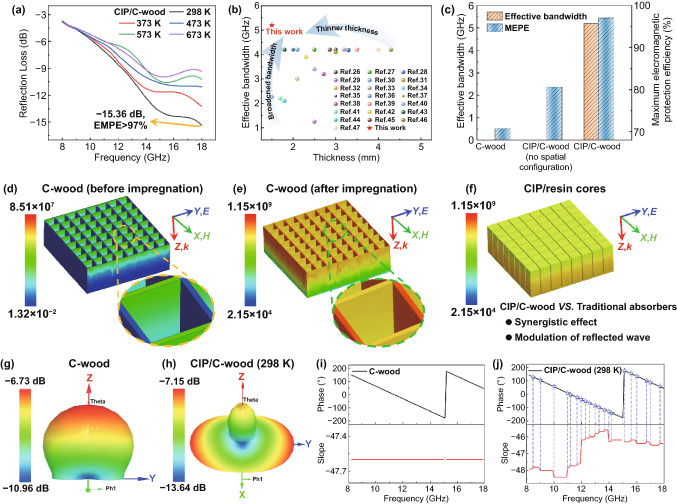
**a** Reflection loss of CIP/C-wood at different temperatures. **b** Comparison of thickness and effective bandwidth to those of previously reported high-temperature microwave absorbing materials. **c** Effective bandwidth and maximum EM protection efficiency (MEPE) of C-wood, CIP/C-wood without spatial configuration and CIP/C-wood. **d** EMW volume loss density of C-wood. **e** EMW volume loss density of structure template for CIP/C-wood. **f** EMW volume loss density of CIP/resin. **g** 3D far-field radiation map of C-wood. **h** 3D far-field radiation map of CIP/C-wood. **i** Phase diagram of C-wood. **j** Phase diagram of CIP/C-wood

The EM protection performance of CIP/C-wood microwave modulator at different temperatures is evaluated by its reflection loss (RL) which is experimentally measured by arch method. As shown in Fig. 3a, CIP/C-wood microwave modulator with a thickness of only 1.5 mm exhibits excellent EM protection performance in the temperature range of 298–673 K, the maximum effective bandwidth (RL less than − 10 dB, EM protection efficiency more than 90%) coverage of 12.8–18.0 GHz and the maximum EM protection efficiency (MEPE) more than 97%. Even when the temperature rises to 673 K, CIP/C-wood microwave modulator still demonstrates over 87.4% (RL < − 9 dB) EM protection efficiency in the frequency range of 13.8–18.0 GHz. To assess the performances of CIP/C-wood microwave modulator more comprehensively, we compare the results with those from representative literature works applied in the temperature range from 298 to 673 K, in terms of thickness and effective bandwidth. As depicted in Fig. 3b, CIP/C-wood microwave modulator demonstrates a superior EM protection capacity at a thinner thickness compared to conventional high-temperature microwave absorbing materials (Table S1) [26–47]. In addition, we find that both effective bandwidth and maximum EM protection efficiency of CIP/C-wood microwave modulator are obviously better than that of C-wood and CIP/C-wood without spatial configuration (Figs. 3c and S7). These results indicate that the excellent EM protection performance of CIP/C-wood microwave modulator not only from intrinsic EM loss of absorbents is also related to the configuration of spaced arrangement for two absorbents.

To elucidate the EM protection mechanisms of CIP/C-wood microwave modulator, a series of EM field simulations are conducted. As shown in Fig. 3d, C-wood, as a structural template, demonstrates an EM loss characteristic with directionality. Specifically, EMW volume loss density parallel to the electric field direction (Y axis) is obviously greater than that perpendicular to the electric field direction (X axis), which reveals that the main EMW absorption mechanism of C-wood is dielectric loss. Furthermore, EMW loss density shows a significant attenuation along the direction of EMW propagation (Z axis), resulting in almost no EMW absorption in the middle and bottom of C-wood. After impregnation of CIP/resin into C-wood channels, EMW volume loss density of C-wood is prominently enhanced, especially in the direction parallel to the magnetic field (Fig. 3e). Although EMW loss density of C-wood still attenuates along the Z axis, the presence of CIP/resin cores improve EMW absorption properties at the bottom of CIP/C-wood microwave modulator. As demonstrated in Fig. 3f, EMW loss density of CIP/resin cores increases along the Z axis, which means that stronger EMW absorption occurs at the bottom of cores. On the other hand, comparing the loss tangent of dielectric and magnetic for C-wood and CIP/resin composite, the significant increase in loss tangent indicates that filling CIP/resin composite into channels of C-wood is an effective way to improve EM loss capability (Fig. S8). Because the introduction of magnetic loss is accompanied by the construction of multi-level interfaces enhancing the dielectric loss. Therefore, the combination of C-wood and CIP/resin achieves a synergistic enhancement in dielectric and magnetic losses.

In addition to excellent intrinsic EM loss capability, CIP/C-wood microwave modulator also demonstrates another EM protection mechanism, radiation deflection. As shown in 3D far-field radiation maps of C-wood and CIP/C-wood (Fig. 3g, h), the intensity of reflected EMW in the Z axis direction is evidently diminished and the angle between the maximum radiation direction and the Z axis visibly increases when the filling medium of C-wood channels is changed from air to CIP/resin. According to electromagnetic transmission theory [10, 12, 48–52], radiation direction of EMW is closely related to transmission phase of EMW and EM properties of media. Figure S9 and S10 shows that the difference in EM parameters between CIP/resin and C-wood is much greater than the difference in EM parameters between air and C-wood in the frequency range of 8.2–18.0 GHz. And these significant differences in EM parameters are fixed in the horizontal direction by spaced arrangement of CIP/resin and C-wood, thus breaking the translation invariance of EM characteristics (Fig. S6b). As a result, different from the continuous phase diagram (consistent slope of curve) of C-wood, multiple discontinuities (inconsistent slope of curve) appear in the phase diagram of CIP/C-wood in the frequency range of 8.2–18.0 GHz (Fig. 3i, j). The radiation direction of EMW controlled by phase is also deflected accordingly. Actually, in the case of small-angle incidence of EMW, obtaining a large-angle reflected EMW is what we expect. As shown in Fig. S11, unabsorbed EMW with a large reflection angle can reduce the probability of multiple EM interference and EM cloaking failure compared to EMW with a small reflection angle.

More gratifyingly, the modulation effect of CIP/C-wood on reflected EMW is not only limited to room temperature environment. Due to the pronounced difference in EM parameters between CIP/resin and C-wood in the temperature range of 298–673 K, discontinuity of EM characteristics in the horizontal direction is maintained even at high temperatures (Figs. S9 and S10). Therefore, multiple discontinuities are observed in the reflection phase diagrams of CIP/C-wood at different temperatures and the corresponding 3D far-field radiation maps show similar reflected EMW modulation properties to those at room temperature (Figs. S12 and S13). Although reflection intensity of EMW along the Z axis increases with temperature, there is still a large portion of unabsorbed EMW that are reflected at large angles.

### Multifunctional Integration

**Fig. 4:**
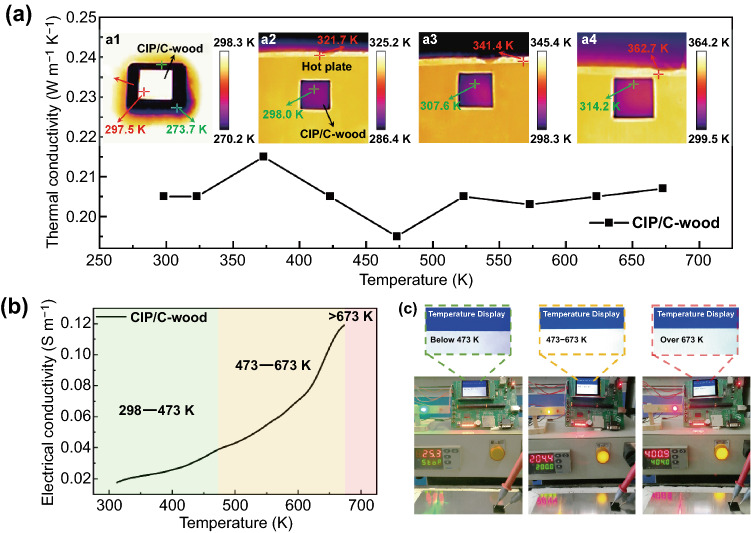
**a** Thermal conductivity of CIP/C-wood microwave modulator at different temperatures. **a1** Thermal infrared image of CIP/C-wood microwave modulator in low-temperature environment. **a2–a4** Thermal infrared images of CIP/C-wood microwave modulator in high-temperature environments. **b** Electrical conductivity of CIP/C-wood microwave modulator at different temperatures. **c** CIP/C-wood as a temperature sensor for monitoring operating temperatures and over-temperature alarm

Besides EM interference, for variable-frequency electronic devices in different operating conditions, their surface temperatures often differ significantly from the surroundings, which would result in a remarkable radiation contrast in thermal images, thus reducing thermal infrared stealth performance of some military and industrial targets [53–59]. It is necessary to blend the protected targets into background in thermal images to dodge infrared detection. It is well known that carbonized wood has good thermal insulation properties, and expansion followed by contraction of CIP/resin in channels of C-wood during the stabilization would destroy part of heat transfer paths. (Fig. S4 and S5). The synergistic effect of structure and ingredients enables CIP/C-wood microwave modulator to exhibit low and stable thermal conductivities in the temperature range of 298 to 673 K (Fig. 4a). Another superiority of CIP/C-wood microwave modulator is that its infrared emissivity of 0.95 is comparable with the value of most backgrounds. Hence, CIP/C-wood microwave modulator could provide good thermal camouflage for protected targets in different surroundings. Here, we use ice placed at room temperature to simulate the case where the target temperature is below the background temperature. A significant radiation contrast between target and background presents in thermal image, but the area covered with CIP/C-wood microwave modulator is basically consistent with background (Fig. 4a1). Moreover, the more general thermal stealth situations, hiding a hot target in a relative cool background, are also simulated at different temperatures. As shown in Fig. 4a2–a4, when the temperature of hot plate increases from 321.7 to 362.7 K, the temperature in area covered with CIP/C-wood microwave modulator only increases from 298.0 to 314.2 K due to good thermal insulation properties, thus effectively weakening the radiation contrast between target and background.

Whether for protected devices or high-temperature EM protection materials, dynamic operating temperatures sometimes pose potential safety risks, like over-temperature. There is a necessity to propose corresponding measures to enhance operational safety. Here, CIP/C-wood microwave modulator shows a monotonic electrical conductivity-temperature characteristic over the temperature range of 298–673 K, which inspires us that this microwave modulator could be used as a temperature sensor for real-time monitoring of working temperature (Fig. 4b). We schematically divide temperature into three ranges, 298–473 K, 473–673 K and > 673 K, corresponding to three color indicators, respectively. Electrical conductivity of CIP/C-wood at different temperatures is integrated into a microcontroller as database for determining the operating temperature. A hot plate is used to simulate the changes in operating temperature, and microcontroller could determine the temperature range of microwave modulator through the data measured by probes in real time, the detailed working principle as depicted in Fig. S14 and Supplementary Notes. In the meantime, indicator and display visualize temperature changes through color variations and text, respectively (Figs. 4c and S15). For example when the working temperature exceeds 673 K, red indicator lights up and displays issues an over-temperature alarm. Actually, commonly used devices already contain microcontroller, display and indicator, thus working temperature monitoring and over-temperature alarm can be realized only by installing two probes on the surface of CIP/C-wood microwave modulator. By this simple method, the operational safety of devices is effectively guaranteed.

## Conclusion

In summary, we demonstrate a bio-inspired CIP/C-wood microwave modulator for high-temperature EM protection, infrared stealth and working temperature monitoring. The spaced arrangement of two microwave absorbents, C-wood and CIP/resin, successfully breaks the translational invariance of electromagnetic characteristics in the horizontal direction, achieving multiple discontinuous phases and large-angle reflected EMW over a wide frequency range. Thus, CIP/C-wood microwave modulator integrated of two EM protection mechanisms concurrently, microwave absorption and radiation deflection, exhibits an effective bandwidth of 5.2 GHz and maximum EM protection efficiency over 97% in the temperature range of 298–673 K with a thickness of only 1.5 mm. Benefiting from the synergistic effect of structure and ingredients, CIP/C-wood microwave modulator shows stable and low thermal conductivities, which could achieve thermal infrared stealth of protected targets in different surroundings. In addition, due to the monotonic electrical conductivity-temperature characteristic, CIP/C-wood microwave modulator could also be employed as a temperature sensor for monitoring operating temperatures and over-temperature alarm. It is believed that this multifunctional CIP/C-wood microwave modulator opens the door for the design and construction of high-efficiency EM protection materials applicable to complex environments.

## Supplementary Information

Below is the link to the electronic supplementary material.

Supplementary file 1 (PDF 1909 kb)
