# Supplementary material for: Bio-Inspired Microwave Modulator for High-Temperature Electromagnetic Protection, Infrared Stealth and Operating Temperature Monitoring
Source: Nanomicro Lett. 2021 Dec 13;14:28. doi: 10.1007/s40820-021-00776-3 (PMC8669058; doi:10.1007/s40820-021-00776-3)
Supplement: Supplementary file 1 — (PDF 1909 kb) [file 40820_2021_776_MOESM1_ESM.pdf]

Supporting Information for

Bio-Inspired Microwave Modulator for High-Temperature Electromagnetic Protection, Infrared Stealth and Operating Temperature Monitoring

Xuan Yang[1], Yuping Duan[1,*], Shuqing Li[2], Huifang Pang[1], Lingxi Huang[1], Yuanyuan Fu[1], Tongmin Wang[1,*]

[1]Key Laboratory of Solidification Control and Digital Preparation Technology (Liaoning Province), School of Materials Science and Engineering, Dalian University of Technology, Dalian 116085, P. R. China

[2]Science and Technology on Power Beam Processes Laboratory, AVIC Manufacturing Technology Institute, Beijing 100024, P. R. China

*Corresponding authors. E-mail: duanyp@dlut.edu.cn (Yuping Duan); tmwang@dlut.edu.cn (Tongmin Wang)

S1 Supplementary Figures

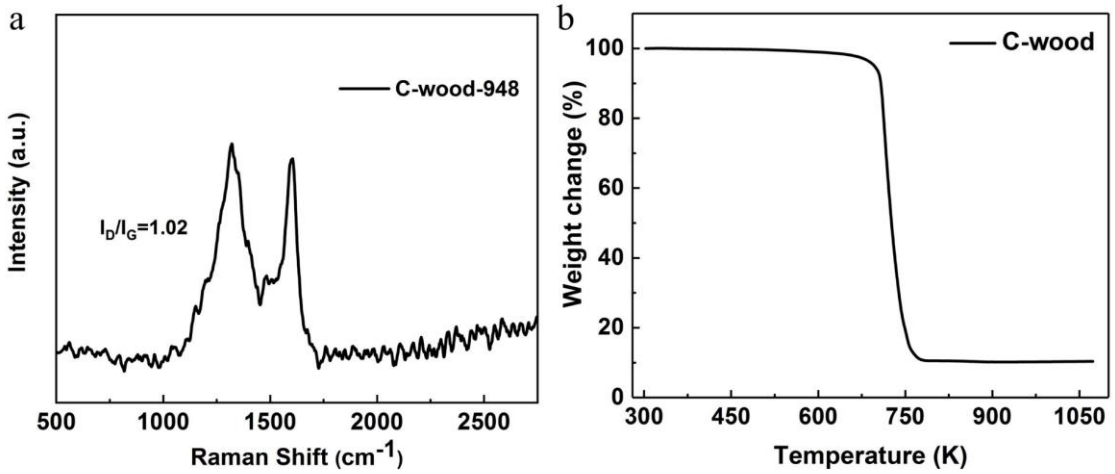

Fig. S1 (**a**) Raman spectrum of C-wood. (**b**) Thermogravimetric analysis of C-wood

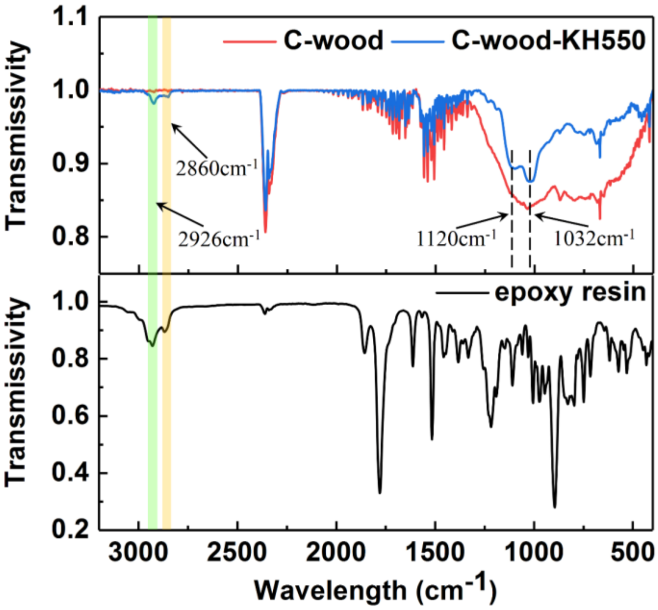

Fig. S2 FTIR spectra of C-wood, C-wood-KH550, and epoxy resin

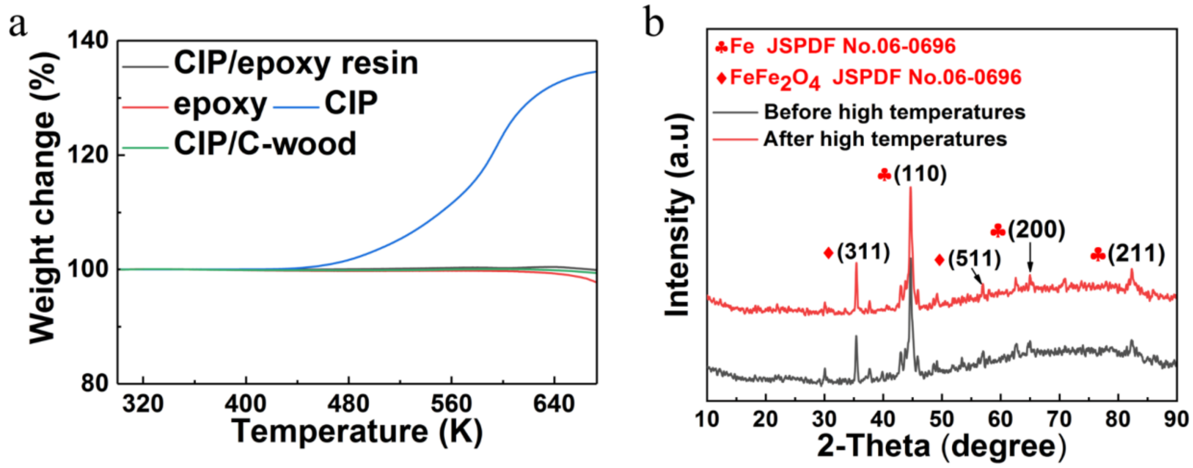

Fig. S3 (**a**) Thermogravimetric analysis curves of CIP/resin, epoxy, CIP and CIP/C-wood. (**b**) XRD patterns of CIP/C-wood before and after high temperatures

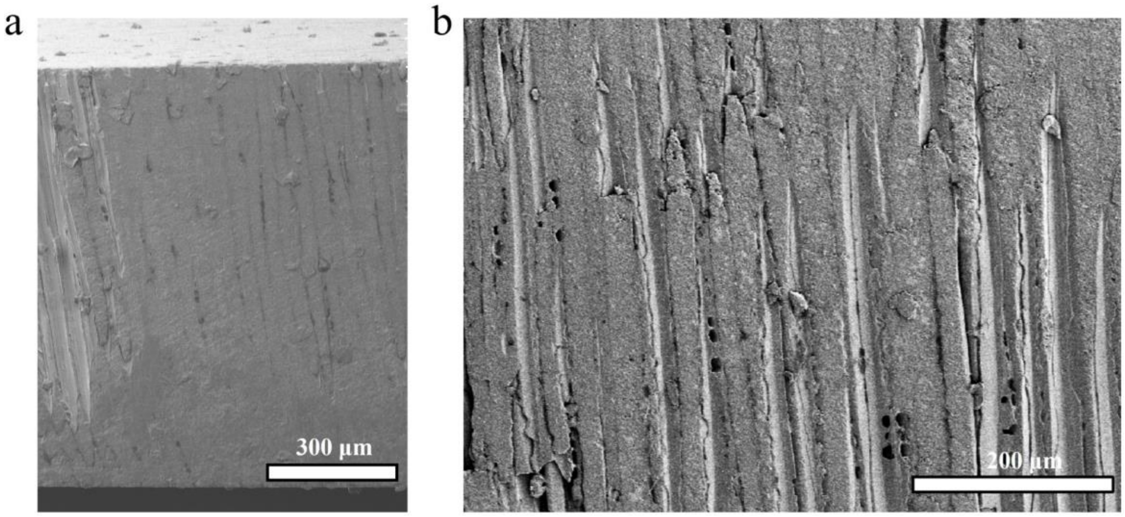

Fig. S4 SEM images of CIP/C-wood before stabilization

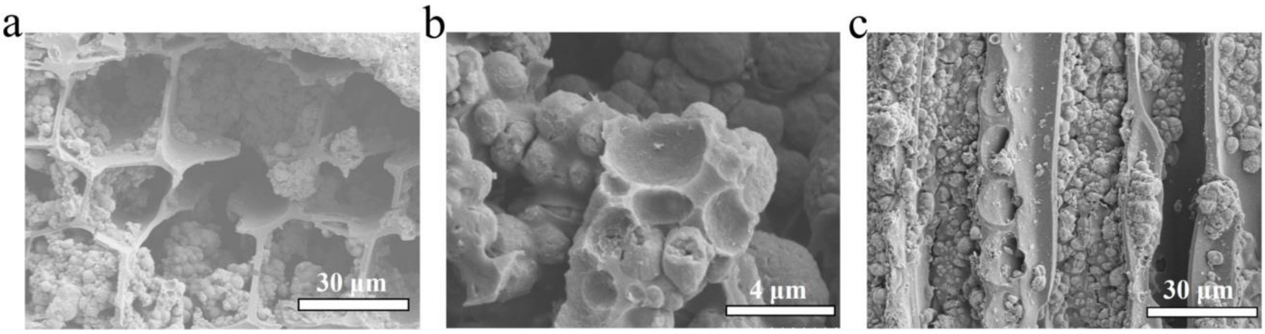

Fig. S5 (**a**) Top-view SEM image of CIP/C-wood after stabilization. (**b**) SEM image of CIP/resin after stabilization. (**c**) Cross-section SEM image of CIP/C-wood after stabilization

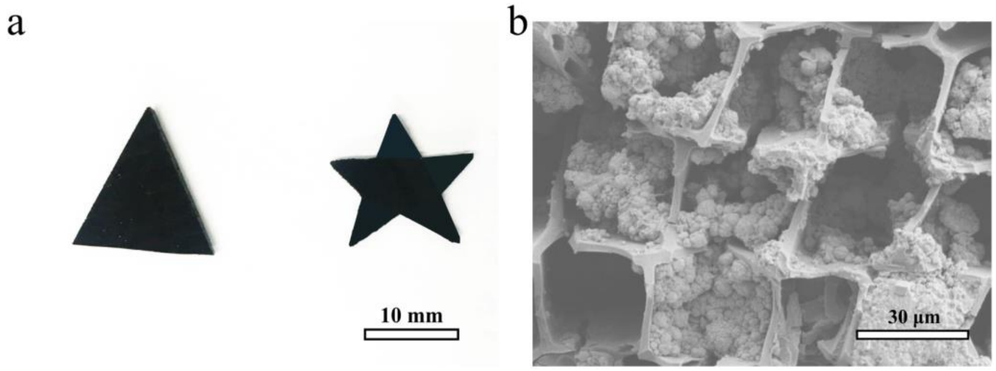

Fig. S6 (**a**) Photograph of CIP/C-wood with different shapes. (**b**) SEM image of CIP/C-wood

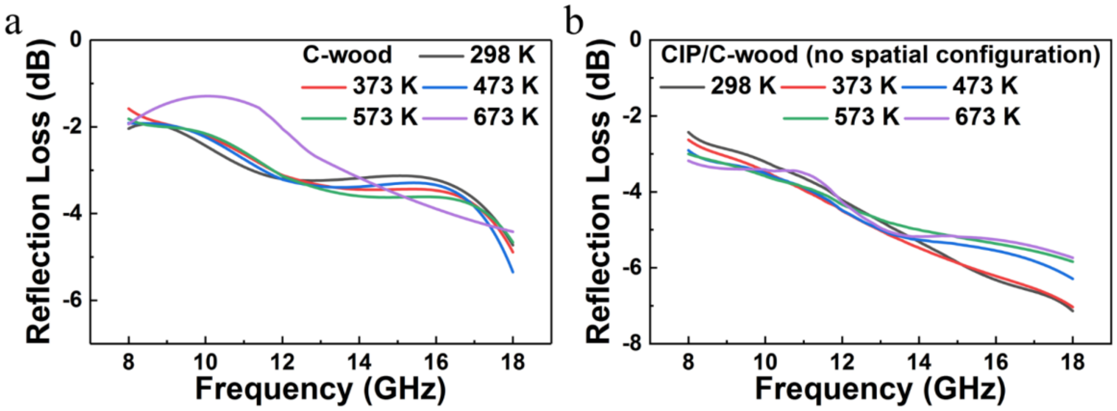

Fig. S7 (**a**) Reflection loss of C-wood. (**b**) Reflection loss of CIP/C-wood without spatial configuration

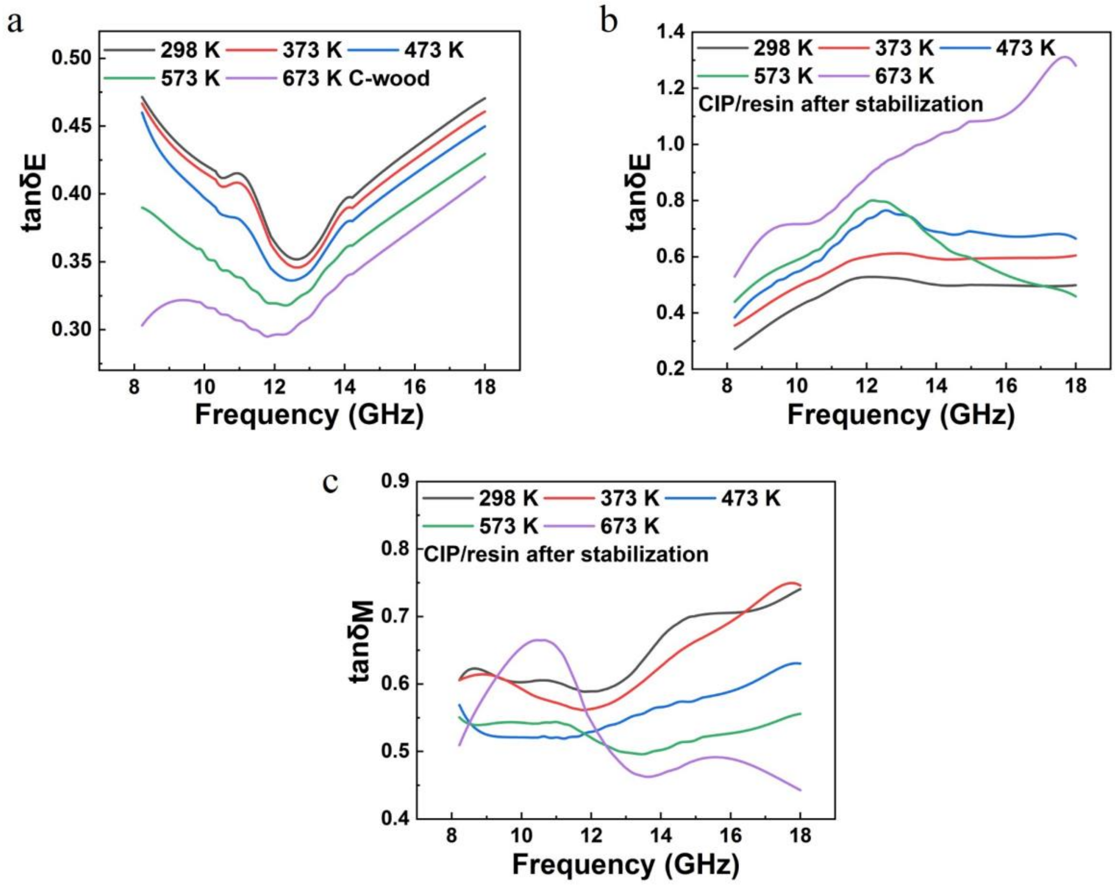

Fig.8 (**a**) Dielectric loss tangent of C-wood. (**b**) Dielectric loss tangent of CIP/resin composite. (**c**) Magnetic loss tangent of CIP/resin composite

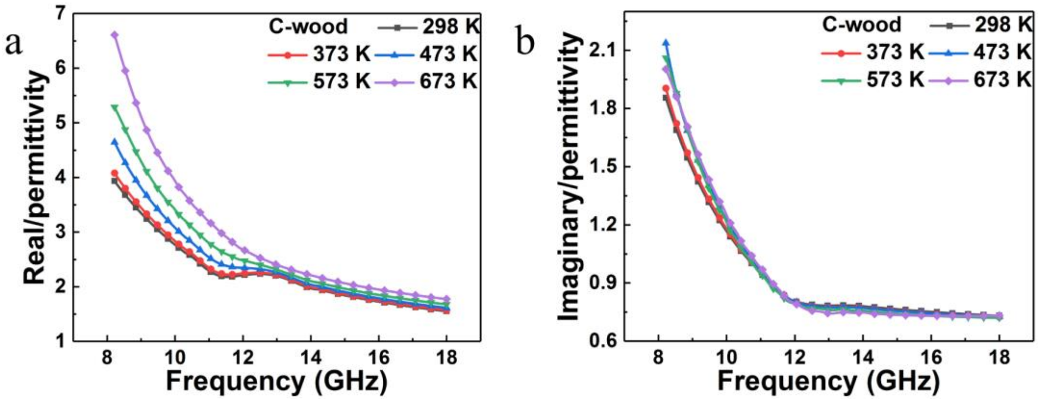

Fig. S9 Electromagnetic parameters of C-wood at different temperatures

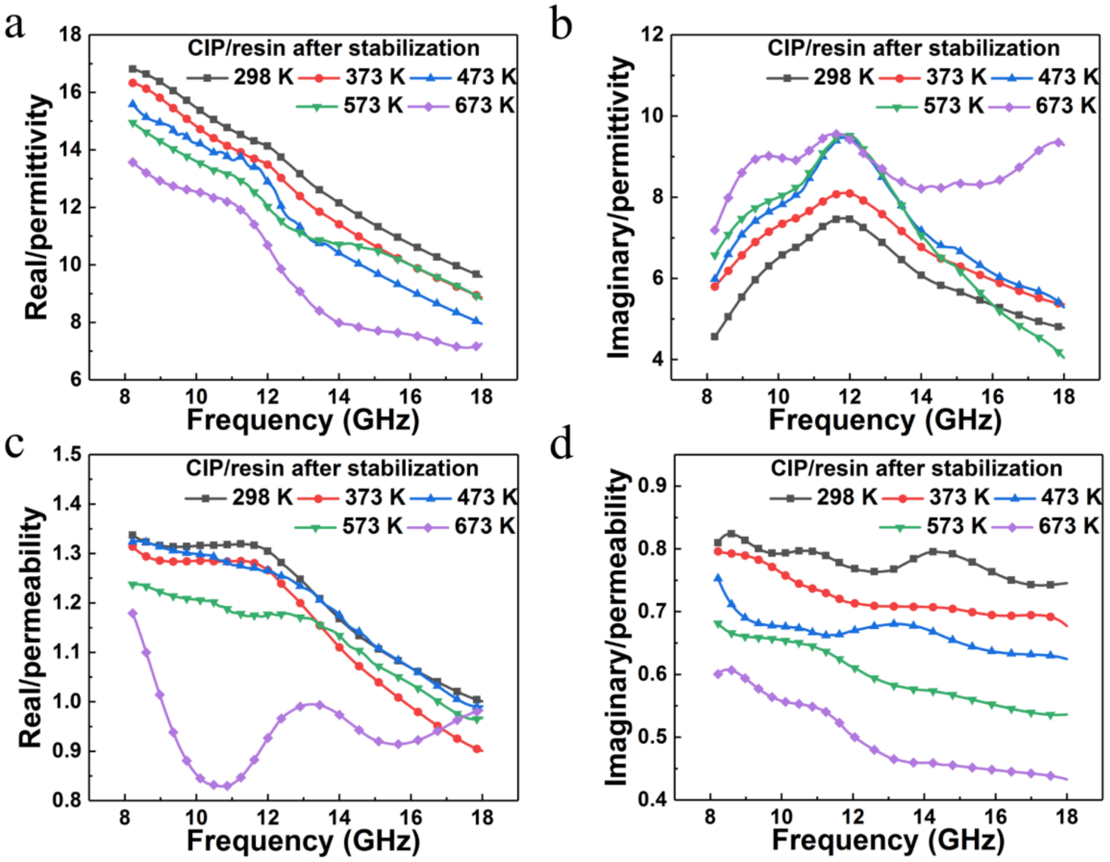

Fig. S10 Electromagnetic parameters of CIP/resin (after stabilization) at different temperatures

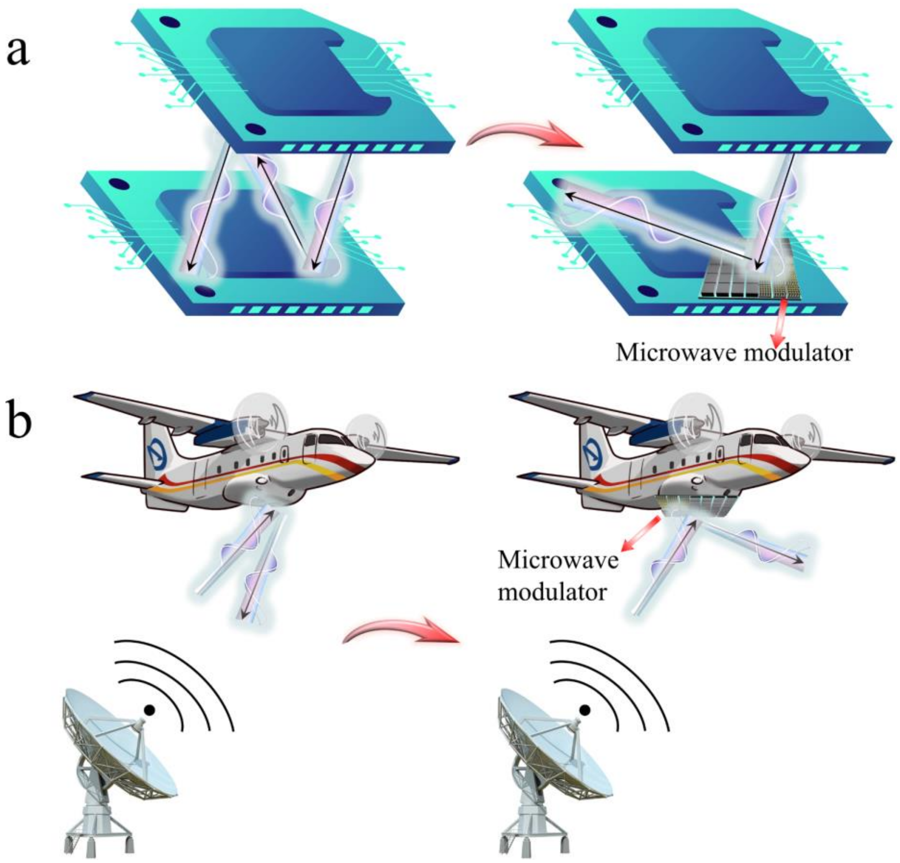

Fig. S11 (**a**) Microwave modulator attenuates electromagnetic interference between adjacent electronic components by changing the direction of reflected wave. (**b**) Microwave modulator enhances the electromagnetic stealth of aircrafts by changing the direction of reflected wave

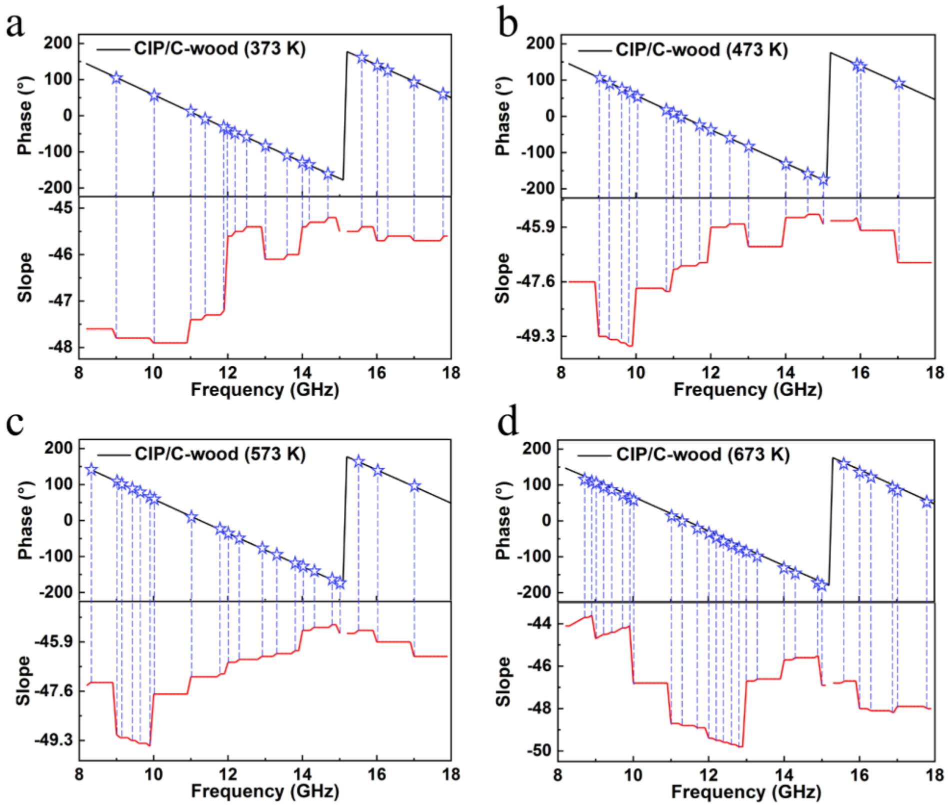

Fig. S12 Phase diagrams of CIP/C-wood at different temperatures

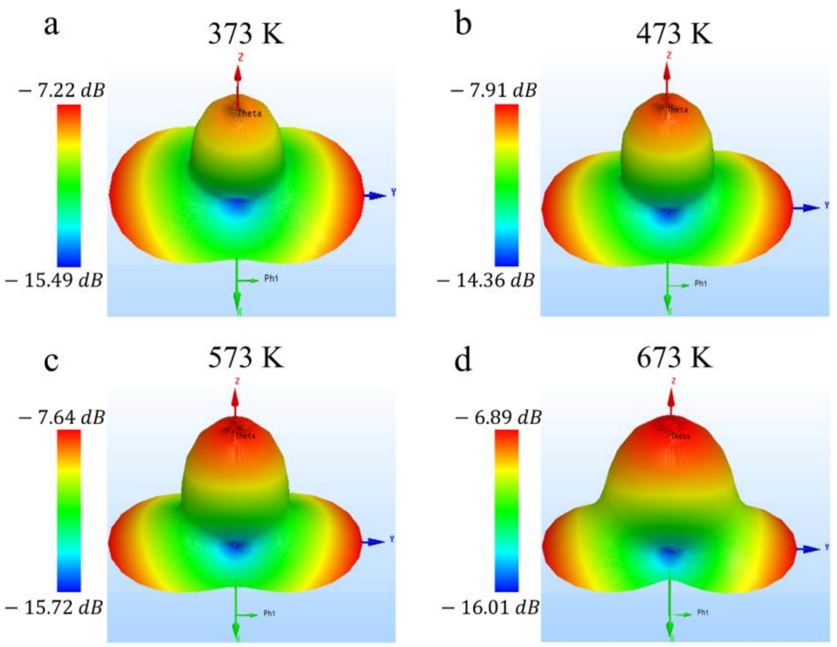

Fig. S13 Far-field radiation maps of CIP/C-wood at different temperatures

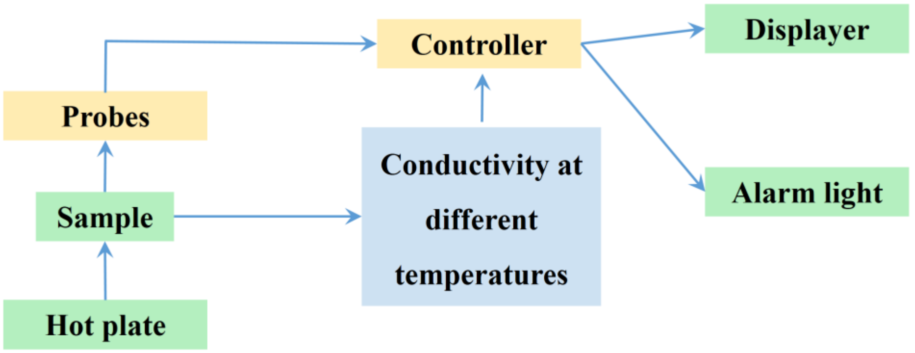

Fig. S14 Working schematic diagram of sensing system for monitoring the working temperature of microwave modulator

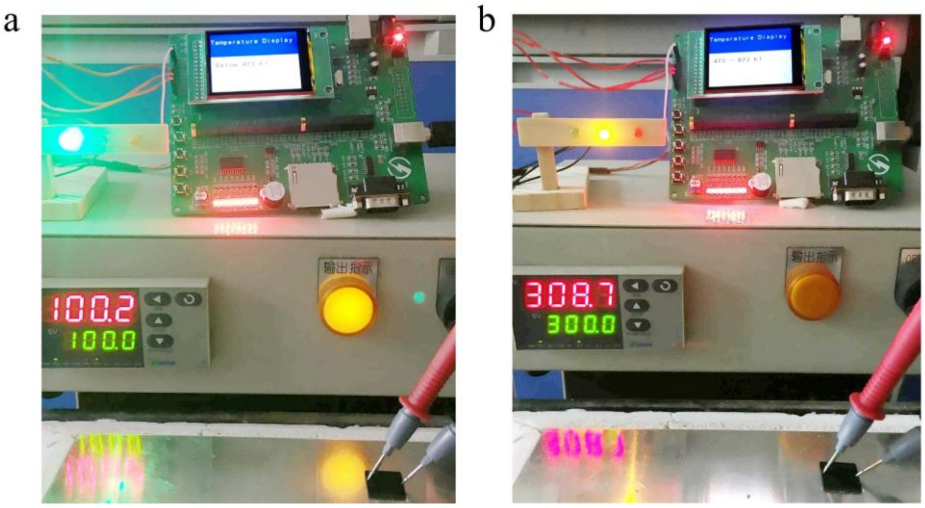

Fig. S15 Photographs of sensing system for monitoring the operating temperature of microwave modulators.

S2 Supplementary Tables

Table S1 Comparison of thickness and maximum effective bandwidth to those of previously reported high-temperature microwave materials

Materials	Thickness (mm)	Maximum effective bandwidth (GHz)	Refs.
LAS/LAS-SiC	3	4.2	[S1]
MWCNTs/SiO_2	2.5	4.2	[S2]
Si_3N_4–SiC/SiO_2	3.3	4.18	[S3]
Zn-doped Al_2O_3	2.5	1.25	[S4]
Ti_3SiC_2/cordierite coatings	1.5	2.26	[S5]
SiC_f/SiC–Al_2O_3	3	4.1	[S6]
SiC powders	2.1	3	[S7]
SiC/SiO_2	3.2	4.2	[S8]
NiO@SiC	2	4.2	[S9]
NiO-SiC	2	4.2	[S10]
Ni-SiC	2.1	4.2	[S11]
Fe-SiC/SiO_2	3.0	4.1	[S12]
CNT–ZnO/glass	2.72	3.2	[S13]
MWCNTs/SiO_2	3.5	4.2	[S14]
ZnO@MWCNTs	2.5	3.4	[S15]
Polyimide/CB and MWCNTs	1.7	2.2	[S16]
graphene/SiC	2.3	3.9	[S17]
La-doped BFO	1.8	4.2	[S18]
Nd-doped BFO	1.8	2.1	[S19]
Al-doped ZnO/$ZrSiO_4$	2.86	4.2	[S20]
RGO/Si_3N_4	4.3	4.2	[S21]
Si_3N_4/SiC Aerogels	4	4.2	[S22]
CIP/C-wood microwave modulator	1.5	5.2	This work

S3 Supplementary Notes

Since the electrical conductivity of CIP/C-wood microwave modulator is monotonically increasing from room temperature to 673 K, this microwave modulator can be used as a temperature sensor for the monitoring of working temperature and over-temperature alarm. In this work, we use microcontroller, resistance probes, hot plate, indicators and CIP/C-wood microwave modulator to build a complete temperature monitoring system to verify the

feasibility of the above design. Hot plate provides a variable temperature environment for microwave modulator, and probes transmit the real-time measured resistance values of CIP/C-wood microwave modulator to microcontroller. Microcontroller converts the resistance into electrical conductivity and compares it with standard values to determine the corresponding operating temperature. In the meanwhile, microcontroller would visualize the working temperature range of microwave modulator through the display and the indicator lights, realizing the monitoring of working temperature and over-temperature alarm. Relevant programs used by microcontroller is shown below:

main.c

```
#include "stm32f10x.h"
#include "lcd.h"
#include "data.h"
#include "stm32f10x_usart.h"
#include "stdlib.h"
#define RB1 GPIO_ReadInputDataBit(GPIOA,GPIO_Pin_0)
#define RB2 GPIO_ReadInputDataBit(GPIOA,GPIO_Pin_8)
#define RB3 GPIO_ReadInputDataBit(GPIOB,GPIO_Pin_1)
#define RB4 GPIO_ReadInputDataBit(GPIOB,GPIO_Pin_2)
#define LED1_ON GPIO_SetBits(GPIOA, GPIO_Pin_5);
#define LED1_OFF GPIO_ResetBits(GPIOA, GPIO_Pin_5);
#define LED2_ON GPIO_SetBits(GPIOA, GPIO_Pin_6);
#define LED2_OFF GPIO_ResetBits(GPIOA, GPIO_Pin_6);
#define LED3_ON GPIO_SetBits(GPIOA, GPIO_Pin_7);
#define LED3_OFF GPIO_ResetBits(GPIOA, GPIO_Pin_7);
uint32_t TimingDelay = 0;
GPIO_InitTypeDef GPIO_InitStructure;
unsigned char data[20];
unsigned char USART2_data;
int Flag=0;
int data_i=0;
void USART_Config(void);
void USART_SendString(int8_t *str);
void Delay_Ms(uint32_t nTime);
void Delay_Ms(uint32_t nTime);
void Key_Init(void);
void LED_Config(void);
void My_Delay(__IO uint32_t nCount);
float getres();
uint8_t Key_Scan(void);
int main(void)
{
   uint8_t key_temp;
float main_temperature=0.0;
   SysTick_Config(SystemCoreClock/1000);
```

```
    LED_Config();
    STM3210B_LCD_Init();
    LCD_Clear(White);
    LCD_SetTextColor(White);
    LCD_SetBackColor(Blue);
    LCD_ClearLine(Line0);
    LCD_ClearLine(Line1);
    LCD_ClearLine(Line2);
    LCD_ClearLine(Line3);
LCD_DisplayStringLine(Line1,"Temperature Display ");
LCD_SetTextColor(Black);
    LCD_SetBackColor(White);
    main_temperature=Get_closest_temperature_data(getres());
    if(main_temperature>673)
    {
        LCD_ClearLine(Line5);
        LCD_DisplayStringLine(Line5," Over 673 K!");
        LED1_ON;LED2_OFF;LED3_OFF;
    }
    if(main_temperature<=673&&main_temperature>=473)
    {
        LCD_ClearLine(Line5);
        LCD_DisplayStringLine(Line5," 473 ~ 673 K!");
        LED1_OFF;LED2_ON;LED3_OFF;
    }
    if(main_temperature<473)
    {
        LCD_ClearLine(Line5);
        LCD_DisplayStringLine(Line5," Below 473 K!");
        LED1_OFF;LED2_OFF;LED3_ON;
    }
}
void LED_Config(void)
{
  RCC_APB2PeriphClockCmd(RCC_APB2Periph_GPIOA | RCC_APB2Periph_AFIO , ENA
BLE);
  GPIO_InitStructure.GPIO_Pin = GPIO_Pin_5 | GPIO_Pin_6 | GPIO_Pin_7;
  GPIO_InitStructure.GPIO_Mode = GPIO_Mode_Out_PP;
  GPIO_InitStructure.GPIO_Speed = GPIO_Speed_50MHz;
  GPIO_Init(GPIOA, &GPIO_InitStructure);
}
void Delay_Ms(uint32_t nTime)
{
```

```
    TimingDelay = nTime;
    while(TimingDelay != 0);
}
void My_Delay(__IO uint32_t nCount)
{
   for(; nCount != 0; nCount--);
}
void USART_Config(void)
{
    GPIO_InitTypeDef  GPIO_InitStructure;
    USART_InitTypeDef USART_InitStructure;

    RCC_APB2PeriphClockCmd(RCC_APB2Periph_GPIOA, ENABLE);
    RCC_APB1PeriphClockCmd(RCC_APB1Periph_USART2, ENABLE);
    GPIO_InitStructure.GPIO_Pin = GPIO_Pin_11;
    GPIO_InitStructure.GPIO_Mode = GPIO_Mode_AF_PP;
    GPIO_InitStructure.GPIO_Speed = GPIO_Speed_50MHz;
    GPIO_Init(GPIOA, &GPIO_InitStructure);
    GPIO_InitStructure.GPIO_Pin = GPIO_Pin_12;
    GPIO_InitStructure.GPIO_Mode = GPIO_Mode_IN_FLOATING;
    GPIO_Init(GPIOA, &GPIO_InitStructure);
    USART_InitStructure.USART_BaudRate = 2400;
    USART_InitStructure.USART_WordLength = USART_WordLength_8b;
    USART_InitStructure.USART_StopBits = USART_StopBits_1;
    USART_InitStructure.USART_Parity = USART_Parity_No ;
    USART_InitStructure.USART_HardwareFlowControl = USART_HardwareFlowControl_
None;
    USART_InitStructure.USART_Mode = USART_Mode_Rx | USART_Mode_Tx;
    USART_Init(USART2, &USART_InitStructure);
    USART_Cmd(USART2, ENABLE);
}
void USART2_IRQHandler(void)
{

    if(USART_GetITStatus(USART2, USART_IT_RXNE) != RESET)
    {
        USART2_data = USART_ReceiveData(USART2);
        if(Flag)
        {
            data[data_i++]=USART2_data;
        }
        if(USART2_data=='+')
        {
```

```
        Flag=1;
        data_i=0;
        data[data_i++]=USART2_data;
      }

    }
}
float getres()
{
   int i=0;
   int j=0;
   char num[4];
   int number;
   float result;
   for(i=1;i<5;i++)
   {
      num[j++]=data[i];
   }
   number=atoi(num);
   switch(data[6]){
      case 0:result=number*1000.0;break;
      case 1:result=number*1.0;break;
      case 2:result=number*10.0;break;
      case 3:result=number*100.0;break;
   }
   result=1000.0/(result*1.5);
   return result;
}
```

data.c

```
#include "data.h"
#include "stdlib.h"
#include "math.h"
float chartofloat(char c[])
{
   int i=0;
   int j=0;
   int k=0;
   int a=0;
   int b=0;
   float d=0.0;
   char inter[5];
   char xiaoshu[10];
   for(i=0;c[i]!='.';)
```

```
    {
        inter[j++]=c[i++];
    }
    i++;
    for(;c[i]!=' ';)
    {
        xiaoshu[k++]=c[i++];
    }
    a=atoi(inter);
    b=atoi(xiaoshu);
    d=b*1.0/strlen(xiaoshu);
    d+=(a*1.0);
    return d;
}
void tempdata_config()
{
    int i=0;
    int j=0;
    int k=0;
    int flag=0;
    int t_i=0;
    int r_i=0;
    char data_temp[6];
    char data_res[10];
    for(i=0;i<368;i++)
    {
        for(t_i=0;table[j]!=' ';)
            data_temp[t_i]=table[j++];
        for(r_i=0;table[j]!=' ';)
            data_res[r_i]=table[j++];
        j++;
        thisdata[i].mytemperature=chartofloat(data_temp);
        thisdata[i].resistivity=chartofloat(data_res);
    }

}
float Get_closest_temperature_data(float input)
{
    float closestdata;
    float t;
    int min_i;
    int i=0;
    closestdata=fabs(input-thisdata[i].resistivity);
```

```
    for(i=1;i<368;i++)
    {
        t=fabs(input-thisdata[i].resistivity);
        if(t<closestdata)
        {
            min_i=i;
            closestdata=t;
        }
    }
    return thisdata[min_i].mytemperature;
}
```

data.h

```
#ifndef __DATA_H
#define __DATA_H
#include "stm32f10x.h"
char table[]= "312.5    0.017830799 \
312.7   0.018021223 \
313.8   0.018198537 \
314.9   0.018304058 \
316   0.018395185 \
317   0.018423321 \
318.1   0.018500221 \
319.1   0.018538994 \
320.1   0.018580133 \
321.1   0.018708612 \
322.1   0.018764894 \
323.1   0.019000649 \
324.2   0.019148066 \
325.2   0.019250628 \
326.2   0.01938242 \
327.2   0.019573715 \
328.2   0.019669467 \
329.2   0.019831747 \
330.2   0.020092582 \
331.1   0.020326313 \
332.2   0.020549928 \
333.1   0.020650799 \
334.1   0.020779599 \
335.1   0.020852691 \
336.1   0.020907467 \
337.1   0.021002441 \
338.1   0.021046932 \
339.1   0.021097128 \
```

340.1 0.021185307 \
341.1 0.021291556 \
342.1 0.021341672 \
343.1 0.021402115 \
344.1 0.021493565 \
345.1 0.021513783 \
346.1 0.021613482 \
347.1 0.021662853 \
348.1 0.021692524 \
349.1 0.021727631 \
350.1 0.021762092 \
351 0.021800172 \
352 0.021865536 \
353 0.021885379 \
354 0.021909718 \
355 0.021927481 \
356 0.021970255 \
357 0.022007008 \
358 0.02208868 \
359 0.022108755 \
360 0.022137045 \
361 0.02216113 \
362 0.022210534 \
363 0.02233026 \
364 0.022360754 \
365 0.022386471 \
365.9 0.02240521 \
366.9 0.022445573 \
367.9 0.022481475 \
368.9 0.022584376 \
369.9 0.022690981 \
370.9 0.02275529 \
371.9 0.022920786 \
372.9 0.023159273 \
373.9 0.023284256 \
374.9 0.02335601 \
375.9 0.02340819 \
376.9 0.023663368 \
377.9 0.023703485 \
378.9 0.023850052 \
379.9 0.023958424 \
380.9 0.024070331 \
381.8 0.024137862 \

382.8 0.024226422 \
383.8 0.024313832 \
384.8 0.024413052 \
385.8 0.024594086 \
386.8 0.024661465 \
387.8 0.024741904 \
388.8 0.02480147 \
389.8 0.024880448 \
390.8 0.024921546 \
391.8 0.025021818 \
392.8 0.025196689 \
393.8 0.025231809 \
394.8 0.025340488 \
395.8 0.025397077 \
396.7 0.025469416 \
397.7 0.025503103 \
398.7 0.025584458 \
399.7 0.025639397 \
400.7 0.025702739 \
401.7 0.025765447 \
402.7 0.025847218 \
403.7 0.025900195 \
404.7 0.025953912 \
405.7 0.026041204 \
406.7 0.026114695 \
407.7 0.026273953 \
408.7 0.026321119 \
409.7 0.026480559 \
410.7 0.026573188 \
411.7 0.026634933 \
412.6 0.026799703 \
413.6 0.027021638 \
414.6 0.027192226 \
415.6 0.027455208 \
416.6 0.027567816 \
417.6 0.027620543 \
418.6 0.028490225 \
419.6 0.028568739 \
420.6 0.02868382 \
421.6 0.029091174 \
422.6 0.02916469 \
423.6 0.029234243 \
424.6 0.029310724 \

425.6 0.029398359 \
426.6 0.029457892 \
427.5 0.029522614 \
428.5 0.029597038 \
429.5 0.029660949 \
430.5 0.029716295 \
431.5 0.029769984 \
432.5 0.029824243 \
433.5 0.029870684 \
434.5 0.029934326 \
435.5 0.030288675 \
436.5 0.030355745 \
437.5 0.030521732 \
438.5 0.030794717 \
439.5 0.030942689 \
440.5 0.031281748 \
441.5 0.031428841 \
442.4 0.031899668 \
443.4 0.032582863 \
444.4 0.033087418 \
445.4 0.033384666 \
446.4 0.033417738 \
447.4 0.033654085 \
448.4 0.033704231 \
449.4 0.033849719 \
450.4 0.033940712 \
451.4 0.034497968 \
452.4 0.034556584 \
453.4 0.034745607 \
454.4 0.034877272 \
455.4 0.035090985 \
456.4 0.035416331 \
457.4 0.035660206 \
458.3 0.035835002 \
459.3 0.035930676 \
460.3 0.036155215 \
461.3 0.036478679 \
462.3 0.036594836 \
463.3 0.03667671 \
464.3 0.036718909 \
465.3 0.036770471 \
466.3 0.036839502 \
467.3 0.036897011 \

468.3 0.036933357 \
469.3 0.037198474 \
470.3 0.037470447 \
471.3 0.037532108 \
472.2 0.03770121 \
473.2 0.038084033 \
474.2 0.038285963 \
475.2 0.039082657 \
476.2 0.03969463 \
477.2 0.040250273 \
478.2 0.040602823 \
479.2 0.040847616 \
480.2 0.040930921 \
481.2 0.041149585 \
482.2 0.041382325 \
483.2 0.04142654 \
484.2 0.041553357 \
485.2 0.041794678 \
486.1 0.041991008 \
487.1 0.042102258 \
488.1 0.0421545 \
489.1 0.04223968 \
490.1 0.04229019 \
491.1 0.042372369 \
492.1 0.04243448 \
493.1 0.042499514 \
494.1 0.042552934 \
495.1 0.042614694 \
496.1 0.042648964 \
497.1 0.04269932 \
498.1 0.042733389 \
499.1 0.04279378 \
500.1 0.042806161 \
501 0.042857343 \
502 0.043062119 \
503 0.043165457 \
504 0.043253175 \
505 0.043385987 \
506 0.043484618 \
507 0.043620965 \
508 0.043978577 \
509 0.044213041 \
510 0.044667584 \

511 0.045004089 \
512 0.045393563 \
513 0.045540285 \
514 0.045633254 \
515 0.045759257 \
516 0.045822697 \
516.9 0.045890368 \
517.9 0.045921657 \
518.9 0.045990245 \
519.9 0.046180981 \
520.9 0.046223598 \
521.9 0.046654077 \
522.9 0.04698117 \
523.9 0.047985172 \
524.9 0.048001044 \
525.9 0.048484215 \
526.9 0.048868446 \
527.9 0.049432764 \
528.9 0.049938576 \
529.9 0.050200641 \
530.9 0.050522752 \
531.8 0.050734749 \
532.8 0.050997983 \
533.8 0.051262966 \
534.8 0.051900482 \
535.8 0.05226308 \
536.8 0.052312934 \
537.8 0.052452262 \
538.8 0.052502716 \
539.8 0.052597707 \
540.8 0.05263242 \
541.8 0.052714093 \
542.8 0.052843582 \
543.8 0.053010652 \
544.8 0.053100912 \
545.8 0.05318085 \
546.7 0.053232922 \
547.7 0.053297054 \
548.7 0.053362127 \
549.7 0.053472898 \
550.7 0.054525836 \
551.7 0.055129877 \
552.7 0.055507924 \

553.7 0.055661993 \
554.7 0.05596673 \
555.7 0.056215516 \
556.7 0.056480838 \
557.7 0.056603662 \
558.7 0.057056694 \
559.6 0.057215817 \
560.6 0.057404049 \
561.6 0.05778813 \
562.6 0.058197556 \
563.6 0.058527995 \
564.6 0.05950847 \
565.6 0.059740965 \
566.6 0.060367007 \
567.6 0.06094359 \
568.6 0.06132896 \
569.6 0.061793112 \
570.6 0.062111807 \
571.6 0.062585338 \
572.6 0.062956101 \
573.6 0.063546943 \
574.6 0.064079114 \
575.5 0.064480822 \
576.5 0.064878347 \
577.5 0.065357635 \
578.5 0.065943401 \
579.5 0.066616924 \
580.5 0.067133776 \
581.5 0.067763891 \
582.5 0.068342641 \
583.5 0.068566411 \
584.5 0.068941107 \
585.5 0.069271143 \
586.5 0.069628217 \
587.5 0.069948565 \
588.5 0.07011641 \
589.5 0.070433523 \
590.4 0.070995121 \
591.4 0.071245227 \
592.4 0.071512123 \
593.4 0.071857651 \
594.4 0.072270742 \
595.4 0.072410771 \

596.4 0.072816172 \
597.4 0.073157193 \
598.4 0.073316204 \
599.4 0.073717707 \
600.4 0.074038197 \
601.4 0.074292258 \
602.4 0.074405291 \
603.4 0.074732084 \
604.4 0.075026825 \
605.3 0.075464745 \
606.3 0.075812819 \
607.3 0.076171427 \
608.3 0.076486121 \
609.3 0.076607829 \
610.3 0.076884096 \
611.3 0.077198687 \
612.3 0.077389316 \
613.3 0.077595556 \
614.3 0.077800443 \
615.3 0.078262649 \
616.3 0.078457185 \
617.3 0.078505425 \
618.2 0.078662372 \
619.2 0.078830075 \
620.3 0.079080451 \
621.2 0.079380925 \
622.2 0.080152614 \
623.2 0.081076892 \
624.2 0.08149917 \
625.2 0.082621748 \
626.2 0.083358885 \
627.2 0.084061597 \
628.2 0.08455376 \
629.2 0.085220617 \
630.2 0.085770004 \
631.2 0.085902851 \
632.2 0.086227798 \
633.1 0.086454251 \
634.1 0.08687289 \
635.1 0.088935745 \
636.1 0.090827054 \
637.1 0.091120261 \
638.1 0.091744326 \

```
639.1   0.092200364 \
640.1   0.092707742 \
641.1   0.093077881 \
642.1   0.094440134 \
643.1   0.098018948 \
644.1   0.099724595 \
645.1   0.099884848 \
646.1   0.101340032 \
647.1   0.101970961 \
648.1   0.104574535 \
649   0.10609365 \
650   0.106441107 \
651   0.107081044 \
652   0.10890809 \
653   0.110073452 \
654   0.110157849 \
655   0.110382768 \
656   0.112854053 \
657   0.113328422 \
658   0.11399607 \
659   0.114393065 \
660   0.114805884 \
661   0.115209458 \
662   0.115678656 \
663   0.116058948 \
663.9   0.11640382 \
664.9   0.117667426 \
665.9   0.118005259 \
666.9   0.118281756 \
667.9   0.118507426 \
668.9   0.118762444 \
669.9   0.1190493   \
670.9   0.119337027 \
671.9   0.119586967 \
672.9   0.119901455 \
673   0.121027038 \
673.1   0.121601882 \
673.1   0.12207448 \
";
typedef struct tempdata{
    float mytemperature;
    float resistivity;
}t_rdata;
```

```
t_rdata thisdata[370];
float chartofloat(char c[]);
void tempdata_config();
float Get_closest_temperature_data(float input);
#endif
```

Supplementary References

[S1] C.H. Peng, P.S. Chen, C.C. Chang, High-temperature microwave bilayer absorber based on lithium aluminum silicate/lithium aluminum silicate-SiC composite. Ceram. Int. **40**(1), 47–55 (2014). https://doi.org/10.1016/j.ceramint.2013.05.101

[S2] W.L. Song, M.S. Cao, Z.L. Hou, J. Yuan, X.Y. Fang, High-temperature microwave absorption and evolutionary behavior of multiwalled carbon nanotube nanocomposite. Scr. Mater. **61**(2), 201–204 (2009). https://doi.org/10.1016/j.scriptamat.2009.03.048

[S3] M. Li, X. Yin, G. Zheng, M. Chen, M. Tao et al., High-temperature dielectric and microwave absorption properties of Si_3N_4–SiC/SiO_2 composite ceramics. J. Mater. Sci. **50**, 1478–1487 (2015). https://doi.org/10.1007/s10853-014-8709-y

[S4] Y. Wang, F. Luo, P. Wei, W. Zhou, D. Zhu, Enhanced dielectric properties and high-temperature microwave absorption performance of Zn-doped Al_2O_3 ceramic. J. Electron. Mater. **44**, 2353–2358 (2015). https://doi.org/10.1007/s11664-015-3787-4

[S5] J. Su, W. Zhou, Y. Liu, Y. Qing, F. Luo et al., High-temperature dielectric and microwave absorption property of plasma sprayed Ti_3SiC_2/cordierite coatings. J. Mater. Sci. Mater. Electron. **27**, 2460–2466 (2016). https://doi.org/10.1007/s10854-015-4046-4

[S6] Y. Mu, W. Zhou, Y. Hu, H. Wang, F. Luo et al., Temperature-dependent dielectric and microwave absorption properties of SiCf/SiC-Al_2O_3 composites modified by thermal cross-linking procedure. J. Eur. Ceram. Soc. **35**(11), 2991–3003 (2015). https://doi.org/10.1016/j.jeurceramsoc.2015.04.016

[S7] H.J. Yang, J. Yuan, Y. Li, Z.L. Hou, H.B. Jin et al., Silicon carbide powders: temperature-dependent dielectric properties and enhanced microwave absorption at gigahertz range. Solid State Commun. **163**, 1–6 (2013). https://doi.org/10.1016/j.ssc.2013.03.004

[S8] X. Yuan, L. Cheng, S. Guo, L. Zhang, High-temperature microwave absorbing properties of ordered mesoporous inter-filled SiC/SiO_2 composites. Ceram. Int. **43**(1), 282–288 (2017). https://doi.org/10.1016/j.ceramint.2016.09.151

[S9] H. Yang, M. Cao, Y. Li, H. Shi, Z. Hou et al., Enhanced dielectric properties and excellent microwave absorption of SiC powders driven with NiO nanorings. Adv. Opt. Mater. **2**(3), 214–219 (2014). https://doi.org/10.1002/adom.201300439

[S10] H.J. Yang, W.Q. Cao, D.Q. Zhang, T.J. Su, H.L. Shi et al., NiO hierarchical nanorings on SiC: enhancing relaxation to tune microwave absorption at elevated temperature. ACS Appl. Mater. Interfaces **7**(13), 7073–7077 (2015). https://doi.org/10.1021/acsami.5b01122

[S11] J. Yuan, H.J. Yang, Z.L. Hou, W.L. Song, H. Xu et al., Ni-decorated SiC powders: enhanced high-temperature dielectric properties and microwave absorption performance. Powder Technol. **237**, 309–313 (2013). https://doi.org/10.1016/j.powtec.2012.12.020

[S12] X. Yuan, L. Cheng, Y. Zhang, S. Guo, L. Zhang, Fe-doped SiC/SiO_2 composites with ordered inter-filled structure for effective high-temperature microwave attenuation. Mater. Des. **92**, 563–570 (2016). https://doi.org/10.1016/j.matdes.2015.12.090

[S13] L. Kong, X. Yin, M. Han, L. Zhang, L. Cheng, Carbon nanotubes modified with ZnO nanoparticles: High-efficiency electromagnetic wave absorption at high-temperatures. Ceram. Int. **41**(3), 4906–4915 (2015). https://doi.org/10.1016/j.ceramint.2014.12.052

[S14] B. Wen, M.S. Cao, Z.L. Hou, W.L. Song, L. Zhang et al., Temperature dependent microwave attenuation behavior for carbon-nanotube/silica composites. Carbon **65**, 124–139 (2013). https://doi.org/10.1016/j.carbon.2013.07.110

[S15] M.M. Lu, W.Q. Cao, H.L. Shi, X.Y. Fang, J. Yang et al., Multi-wall carbon nanotubes decorated with ZnO nanocrystals: mild solution-process synthesis and highly efficient microwave absorption properties at elevated temperature. J. Mater. Chem. A **2**, 10540–10547 (2014). https://doi.org/10.1039/C4TA01715C

[S16] H. Wang, D. Zhu, W. Zhou, F. Luo, Electromagnetic and microwave absorbing properties of polyimide nanocomposites at elevated temperature. J. Alloys Compd. **648**, 313–319 (2015). https://doi.org/10.1016/j.jallcom.2015.07.006

[S17] M. Han, X. Yin, W. Duan, S. Ren, L. Zhang et al., Hierarchical graphene/SiC nanowire networks in polymer-derived ceramics with enhanced electromagnetic wave absorbing capability. J. Eur. Ceram. Soc. **36**(11), 2695–2703 (2016). https://doi.org/10.1016/j.jeurceramsoc.2016.04.003

[S18] Y. Li, M.S. Cao, D.W. Wang, J. Yuan, High-efficiency and dynamic stable electromagnetic wave attenuation for La doped bismuth ferrite at elevated temperature and gigahertz frequency. RSC Adv. **5**(94), 77184–77191 (2015). https://doi.org/10.1039/C5RA15458H

[S19] Y. Li, X. Fang, M. Cao, Thermal frequency shift and tunable microwave absorption in $BiFeO_3$ family. Sci. Rep. **6**, 24837 (2016). https://doi.org/10.1038/srep24837

[S20] L. Kong, X. Yin, Q. Li, F. Ye, Y. Liu et al., High-temperature electromagnetic wave absorption properties of ZnO/$ZrSiO_4$ composite ceramics. J. Am. Ceram. Soc. **96**, 2211–2217 (2013). https://doi.org/10.1111/jace.12321

[S21] Z. Hou, X. Yin, H. Xu, H. Wei, M. Li et al., Reduced graphene oxide/silicon nitride composite for cooperative electromagnetic absorption in wide temperature spectrum with excellent thermal stability. ACS Appl. Mater. Interfaces **11**(5), 5364–5372 (2019). https://doi.org/10.1021/acsami.8b20023

[S22] Z. Cai, L. Su, H. Wang, M. Niu, L. Tao et al., Alternating multilayered Si_3N_4/SiC aerogels for broadband and high-temperature electromagnetic wave absorption up to 1000°C. ACS Appl. Mater. Interfaces **13**(14), 16704–16712 (2021). https://doi.org/10.1021/acsami.1c02906
